# Theodore Menges, B.S., A.M., D.D.S.

**Published:** 1900-07

**Authors:** 


### Obituary.

### THEODORE MENGES, B.S., A.M., D.D.S.

DR. MENGES died June 1, 1900, after an operation for appendicitis.

The unexpected death of Dr. Menges has brought a feeling of sorrow, not alone to those of his family and intimate associates, but to a large circle covering a wide extent of country. He came into dentistry late in his active life. Having been an educator in the West for many years, he accidentally became interested in dental education, and was for some time personally and financially connected with the American College of Dental Surgery of Chicago. He devoted all his energies to that institution, and finally brought about a union of this school with the Northwestern University Dental School. He fully demonstrated his great business ability in bringing the American College of Dental Surgery up to a high standard of excellence, and this he continued after the union with the Northwestern University, and it was mainly through his exertions, aided by a corps of superior instructors, that this college to-day stands among the leading dental schools of the country, both in point of number of students and training.

He devoted his life and means to the advancement of the dental profession. With some men this would not mean much, but with Dr. Menges it resulted in work day and night, never seeming to weary and never willing to rest while something was to be done to raise the standard of his school and the profession.

He became a member of the National Association of Dental Faculties, through his connection with the American College of Dental Surgery, and continued as delegate after the union with the Northwestern. Those connected with that body soon discovered they had as a fellow-member a born leader of men. His energy was unflagging, and through word and pen he endeavored to carry out his ideas, and that with a force that always commanded a respectful hearing, though not always with assent. He was made chairman of the executive committee of that body in 1899, accepting it, as he informed the writer, because he saw in this most important committee an opportunity for work.

The result of this work was soon made manifest. In his opinion there were many weak schools applying for membership, and some already in the fold that had no right to be considered satisfactory dental colleges. He pushed his investigations in this direction to the fullest extent, and was busily occupied in this field of labor when stricken by death.

While many will honestly differ from the course he pursued, no one but must accord to him credit for the highest probity and an ever-indwelling desire that dental education should be relieved of all the defects with which it is contaminated. Up to a certain limit the writer fully sympathized with this work, and was constantly brought into close relation with his aspirations.

He was courageous, oftentimes to the point of rashness, and this was markedly manifest in his last sickness in refusing at last to take an anæsthetic in the surgical operation to be performed, one of the most serious humanity is called upon to endure.

His death came to the writer as one of the most serious blows of his professional life. Dr. Menges was looked upon as one able and willing to sustain the highest standard possible in dental education. He would not listen to any compromise in this direction, and had he lived he would undoubtedly have created in the National Association of Dental Faculties a sentiment of far-reaching character. Most men are influenced by some ulterior selfish motive, but this did not belong to Dr. Menges. His eye was ever turned to the light as he saw it, and with his impetuous nature obstacles were as nothing to its attainment. Such men are rare, and because of that rarity their departure causes a break in the ranks not possible to at once fill.

It is not difficult to do justice to such a character. His life was an open book to be read of men, but there was an undercurrent that few were privileged to see and feel. He was warm in his friendships and frank in his confidences, and the writer feels, as he parts with him upon the border-land of life, that he has lost one of the truest of companions, one of the most energetic of the true-hearted men devoted to dentistry, and one whose name will be enshrined in his memory among the few faithful devotees to that standard which leads unerringly to a higher professional life.

